# Antioxidant Capacity and Phenolic Content of *Caesalpinia pyramidalis* Tul. and *Sapium glandulosum* (L.) Morong from Northeastern Brazil

**DOI:** 10.3390/molecules16064728

**Published:** 2011-06-07

**Authors:** Carlos Henrique Tabosa Pereira da Silva, Tadeu José da Silva Peixoto Sobrinho, Valérium Thijan Nobre de Almeida e Castro, Danielle da Cunha Amaral Lima, Elba Lúcia Cavalcanti de Amorim

**Affiliations:** 1 ASCES College, Caruaruense Association of Higher Education, 55016-400, Caruaru-PE, Brazil; 2 Department of Pharmaceutical Sciences, Health Sciences Center, Federal University of Pernambuco, 50740-521, Recife-PE, Brazil; Email: tadeu1903@yahoo.com.br (T.J.S.P.S.); valerium_castro@hotmail.com (V.T.N.A.C.); daniellecalima@gmail.com (D.C.A.L.); elba@ufpe.br (E.L.C.A)

**Keywords:** *Caesalpinia pyramidalis*, DPPH assay, FIC assay, Phenolic compounds, *Sapium glandulosum*

## Abstract

The aims of this study were to quantify the phenolic content and evaluate the antioxidant potential of extracts from the bark and leaves of *C. pyramidalis* and *S. glandulosum*. The total phenolic content (TPC) and total tannin content (TTC) were determined using the Folin-Ciocalteu method, and the total flavonoids content (TFC) was measured *via* complexation with aluminum chloride. The antioxidant activity was evaluated with DPPH (2.2-diphenyl-1-picrylhydrazyl) and FIC (ferrous ion chelating) assays. The TPC ranged between 135.55 ± 9.85 and 459.79 ± 11.65 tannic acid equivalents (TAE) in mg/g material (mg TAE/g). The leaves of both species contained high levels of tannins and flavonoids. The crude ethanol extracts (CEE) from the bark of *C. pyramidalis* showed high antioxidant activity when compared to ascorbic acid and rutin, whereas the CEE from the leaves was more efficient in chelating ferrous ions. *C. pyramidalis* had very high phenolic content and anti-radical activity, which indicates a need for further studies aimed at the purification and identification of compounds responsible for the antioxidant activity.

## 1. Introduction

In Brazil, several research groups have evaluated the diversity of the medicinal species from different biomes. However, the therapeutic potential of the Caatinga biome was largely unevaluated [1,2]. It was believed that there was little to be discovered, but these initial beliefs have been disproved, and recent research has focused on the exploration and discovery of new drugs from that source [3,4].

*Caesalpinia pyramidalis* Tul. (Caesalpiniaceae) and *Sapium glandulosum* (L.) Morong (Euphorbiaceae) are species widely found in this biome, but their medical applications are still restricted to traditional communities. These species are also commonly used for other purposes, such as a wood fuel and in joinery [5]. Medicinally, *C. pyramidalis* is used as an expectorant, aphrodisiac, to treat bronchitis, respiratory infections, influenza, asthma, gastritis, colic, fever, heartburn, flatulence, diarrhea, collision, injury, diabetes and stomach aches [6]. The decoction of the stem bark of some species of *Sapium* is used internally as an abortive and purgative and externally to treat ulcers [7]. The isolation of various secondary metabolites, especially polyphenols and terpenoids. from *C. pyramidalis* was reported, but to the best of our knowledge, no chemical profile of the specie *S. glandulosum* has been published.

Based on their traditional uses in folk medicine, several thematic groups have been consolidated and form the basis of new fields, such as ethnopharmacology, that focus on gathering knowledge about the natural resources used in traditional folk medicine [8,9]. Among the various questions investigated in this field, the potential reduction of the time needed for the discovery of new drugs to treat diseases that already have alternative therapies or for those disease without known treatments is the most promising. Although this ethnopharmacological approach is potentially beneficial from the standpoint of pharmacology and toxicology, more in-depth ethical investigations are necessary [10].

The principal focus of ethnopharmacological research today is related to the discovery of new antioxidants [11,12]. Antioxidants inhibit the formation of damaging reactive oxygen species in the body [13]. Antioxidants can also inhibit the peroxidation of biological molecules by chelating transition metals that generate hydroxyl radicals through the Haber-Weiss and Fenton reactions [14]. Phenolic compounds, represented mainly by tannins and flavonoids, stand out as the major group of natural antioxidants. They act as efficient scavengers of free radicals and, due to their ability to act as hydrogen donors, they interrupt oxidative chain reactions [15,16]. The assessment of antioxidant activity has been researched, and different methodologies have been proposed [17,18]. In addition to these assessments, quantitative analysis of the substances responsible for these effects, including plant phenolic compounds, are needed [19,20,21].

Given the therapeutic potential of these plants, the objectives of this paper were to evaluate the content of phenolic compounds, including total phenolic content (TPC), total tannin content (TTC), and total flavanoid content (TFC) and the antioxidant activity (DPPH and FIC assay) of crude ethanol extracts of the bark and leaves of *C. pyramidalis* and *S. glandulosum*.

## 2. Results and Discussion

### 2.1. Phenolic Compound Content

The levels of phenolic compounds obtained from the crude ethanol extracts (CEE) of the bark and leaves of *C. pyramidalis* and *S. glandulosum* are presented in Table 1.

**Table 1:** Total phenolic content (TPC), total tannin content (TTC) and total flavonoid content (TFC) of *Caesalpinia pyramidalis* Tul. and *Sapium glandulosum* (L.) Morong.

Species/Popular name	Part used	TPC (mg TAE/g)	TTC (mg TAE/g)	TFC (mg RE/g)
*Caesalpinia pyramidalis* Tul.Catingueira	Bark	160.82 ± 8.11 a	61.36 ± 2.85 a	114.55 ± 3.34 a
	Leaves	459.79 ± 11.65 b	284.22 ± 4.85 b	370.40 ± 12.80 b
*Sapium glandulosum* (L.) Morong. Burra leiteira	Bark	135.55 ± 9.85 c	ND	107.34 ± 3.05 a
	Leaves	280.68 ± 6.36 d	125.97 ± 9.30 c	312.56 ± 7.34 c

TPC and TTC = milligrams of tannic acid equivalents per gram of dry extract (mg TAE/g). TFC = milligrams of rutin equivalent per gram of dry extract (mg RE/g). ND: not detected; Means followed by the same letter in a column do not differ statistically (n = 6; p < 0.05).

In all cases, significant differences were observed between the different parts of the same species (p < 0.05). However, the phenolic compound content was higher in the leaves. Comparing across species, *C. pyramidalis* showed higher levels of TPC, TTC, and TFC than *S. glandulosum*, with the CEE of the leaves of *C. pyramidalis* showing the most significant results. Unexpectedly, the bark of *S. glandulosum* did not contain tannins. This plant typically utilizes the ability to precipitate proteins of this class to make the plant itself unpalatable, thus reducing predation by herbivores and micro-organisms [22,23,24]. As predicted, the Pearson correlation test showed a significant relationship between TPC × TTC (r = 0.9894), although this was not statistically correlated with TFC.

One explanation for the differing levels of phenolic compounds in the bark and leaves of both species is that some metabolites may be floating in the organs of these plants [25,26]. Monteiro *et al*. [27] studied *C. pyramidalis* and found 29.60 ± 12.69 mg/g TPC and 24.72 ± 11.53 mg/g TTC in the bark, which differs significantly from the values found in this study. The authors reported that they did not determine the content in the leaves because they were unable to obtain the plant material during the collection period [27].

### 2.2. Antioxidant Activity

#### 2.2.1. Radical Scavenging Activity (DPPH Assay)

**Figure 1:**
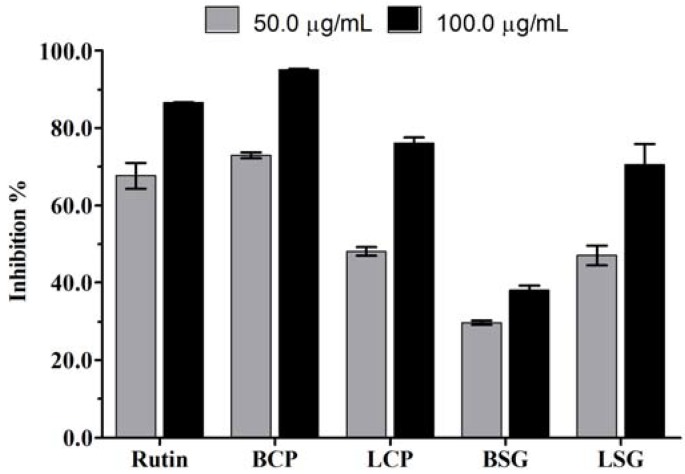
Radical scavenging activity of the CEE of the bark and leaves of *C.pyramidalis* Tul. and *S. glandulosum* (L.) Morong. compared with rutin. BCP = bark of *C. pyramidalis*; LCP = leaves of *C. pyramidalis*; BSG = bark of *S. glandulosum*; LSG = leaves of *S. glandulosum*.

Differences were observed in the radical scavenging activity both between the different species and between the different parts of the same species (Table 2). Of the samples tested, the CEE of the bark of *C. pyramidalis* presented the lowest concentration of extract needed to reduce free radicals by 50% (IC_50_ = 16.98 ± 1.34 µg/mL), and this value was statistically higher than rutin. Although the CEE of the bark of *C. pyramidalis* was more efficient in removing free radicals than the CEE of the leaves, the CEE of *S. glandulosum* leaves was more efficient than the CEE of the bark. The assessment of the percentage of antioxidant activity at 100 µg/mL showed that the CEE of the bark of *C. pyramidalis* was statistically higher than rutin at the same concentrations (Figure 1).

**Table 2:** Radical scavenging activity (DPPH assay), ascorbic acid equivalent antioxidant capacity (AEAC) and chelating activity (FIC assay) of the bark and leaves of *C. pyramidalis* Tul. and *S. glandulosum* (L.) Morong.

Control/Species Name	Part used	Yield	Antioxidant activity (AOA)		
			DPPH free radical scavenging		FIC assay IC_50_ (µg/mL)
			IC_50_ (µg/mL)	AEAC (mg AA/100 g)	
Rutin	-	-	22.96 ± 1.99 b	70.65 ± 6.09 a	-
EDTA	-	-	-	-	15.26 ± 0.58 a
*Caesalpinia pyramidalis* Tul. Catingueira	Bark	11.54%	16.98 ± 1.34 a	95.40 ± 7.75 b	527.44 ± 37.73 b
	Leaves	26.78%	38.93 ± 0.71 c	41.41 ± 0.76 c	62.49 ± 10.77 c
*Sapium glandulosum* (L.) Morong. Burra-leiteira	Bark	16.30%	183.45 ± 7.60 d	8.80 ± 0.37 d	2152.98 ± 490.38 d
	Leaves	24.28%	58.55 ± 6.35e	34.11 ± 2.39 c	127.10 ± 9.44 e

Means followed by the same letter in a column do not differ significantly (n = 6, p <0.05).

According to Melo *et al*. [28], antioxidant activity can be classified based on the performance of the crude extract: I − good activity (IC_50_ < 69 µg/mL, up to three times the inhibitory concentration of the standard); II − moderate activity (69 µg/mL < IC_50_ < 161 µg/mL, between three and seven times the inhibitory concentration of the standard); III − low activity (IC_50_ > 161 µg/mL, exceeding seven times the inhibitory concentration standard). Using this classification, three of the CEEs showed good activity, and only the CEE of the bark of *S. glandulosum* showed low activity (Table 2).

Some studies have suggested that extracts or compounds that exhibit activity against the DPPH free radical can be considered as primary antioxidants, since these compounds act as electron donors and interrupt the chain reactions [29,30,31]. Our results showed that the CEE of the bark of *C. pyramidalis* has high radical scavenging capacity and can be used to inhibit the oxidation of vital substances by reactive oxygen species, possibly by acting as a primary antioxidant. In a recent study, Alviano *et al*. [32] used the DPPH assay and found an IC_50_ of 15.2 ± 1.0 µg/mL for the aqueous extract of leaves from *C. pyramidalis*. This result implies that this compound is more efficient than reported here; however, the extraction liquid used may have influenced the activity. Another factor influencing the results is that water restricts the extraction of various compounds of different polarity, which may contribute to the increased concentration of antioxidants in the aqueous extract.

Recent studies have shown that phenolic compounds, such as tannins and flavonoids, may be linked to the antioxidant activity of many plants [33,34]. This relationship was documented by Rumbaoa *et al*. [35], who found a negative correlation between the TPC and the antioxidant activity (DPPH, r = −0.826; FIC, r = −0.800) of five varieties of *Ipomoea batatas* (L.) Lam (Convolvulacae), indicating that when the TPC is higher, the inhibitory concentration will be lower. Luo *et al*. [36] assessed the TPC and antioxidant activity of various fractions of the aqueous extract of *Dracaena cambodiana* Pierre ex. Gagnep (Asparagaceae) and observed that the ethyl acetate fraction contained higher levels of phenols and showed the highest radical scavenging ability, indicating that this species can be used as a natural antioxidant. a strong relationship between the total antioxidant capacity with TPC (R^2^ = 0.9220) and TFC (R^2^ = 0.8120) of the ethyl acetate fractions extracted from six species of *Ficus* (Moraceae) was also exhibited and the butanol fractions showed that good relations with TPC (R^2^ = 0.604) and TFC (R^2^ = 0.678), suggesting that these groups are responsible for the antioxidant capacity of the species [37]. However, a study by Malenčić *et al*. [38] on the acetone extract of twenty hybrids of *Glycine max* (L.) Mer. (Fabaceae) showed no correlation between antioxidant activity and TFC, nevertheless, observed for polyphenols (r = 0.6696), tannins (r = 0.7465) and proanthocyanidins (r = 0.6538). However, some of these compounds are not always related to antioxidant activity [39,40,41,42], as assessed by the Pearson correlation test between the inhibitory concentrations (IC_50_) and TPC (r = −0.4618), TTC (r = −0.5716) and TFC (r = −0.4307).

#### 2.2.2. Chelating Ability (FIC Assay)

**Figure 2:**
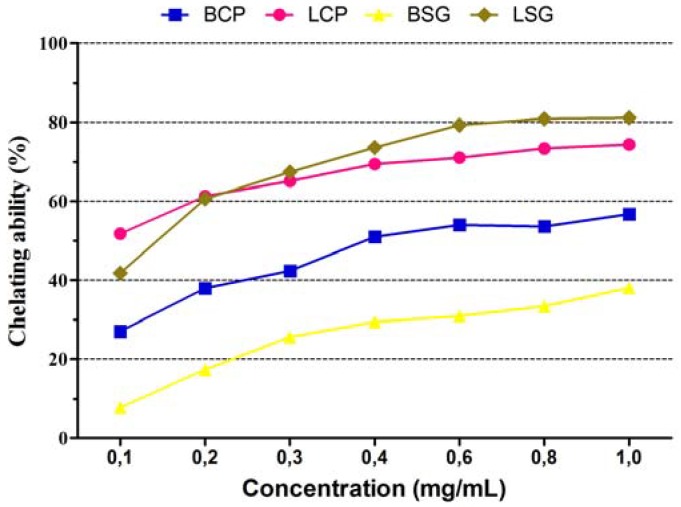
The chelating capacity of the CEE of the bark and leaves of *Caesalpinia pyramidalis* Tul. e *Sapium glandulosum* (L.) Morong. BCP = bark of *C. pyramidalis*; LCP = leaves of *C. pyramidalis*; BSG = bark of *S. glandulosum*; LSG = leaves of *S. glandulosum*.

Figure 2 shows that the chelating capacity of extracts increased proportionally to the concentration. The most promising result was found for the CEE of the leaves of *C. pyramidalis* (Table 2). The CEE from the bark of *S. glandulosum* showed low chelator activity. A Pearson correlation test did not reveal any relationship between FIC and the phenolic content.

The presence of transition metals in biological systems can catalyze the Haber-Weiss and Fenton reactions, resulting in the generation of hydroxyl radicals [14]. However, these transition metals can be chelated by antioxidants, resulting in the suppression of the generation of OH- and an inhibition of the peroxidation of biological molecules. The high FIC capacity of the CEE of *C. pyramidalis* leaves suggests that they contain higher amounts of ligands to compete with ferrozine and prevent the generation of hydroxyl radicals. Kostyuk *et al*. [43] reported that flavonoids bind to metal ions and are much less prone to oxidation than the free compounds in the presence of superoxide.

## 3. Experimental

### 3.1. Plant Material

The stem barks are the parts typically indicated in ethnopharmacological surveys, with the leaves being collected as a possible ecological alternative to the use of non-renewable parts. Samples of the bark and leaves of *C. pyramidalis* and *S. glandulosum* were collected on May 5th and 6th of 2009 from a remnant of Caatinga within the Climatological Station of the Instituto Agronômico de Pernambuco-IPA (08°14’18.2”S and 35°54’57.1”W). The region has a Bsh climate (hot semi-arid), lies at an altitude of 537 m above sea level and has an average precipitation below 700 mm/year [44]. In the same period, the voucher specimens were prepared and incorporated in the Herbarium UFP Geraldo Mariz, Department of Botany, Federal University of Pernambuco, under the number 60.195 (*C. pyramidalis*) and 60.196 (*S. glandulosum*).

### 3.2. Reagents, Reference Standards and Equipment

Ethanol (Vetec, 99.5%) was used as the solvent to extract the samples. To determine the TPC and TTC, anhydrous sodium carbonate (Vetec, 99.5%) and Folin-Ciocalteu phenol reagent (Fluka, 2 N) were used. Glacial acetic acid (Merck, 100%), aluminum chloride hexahydrate (Honeywell, 99%), and pyridine (Vetec, 99%) were used to quantify the flavonoid content. For the DPPH assay, 2.2-diphenyl-1-picrylhydrazyl (Aldrich, 95%) was used. Methanol (Vetec, 99.8%), ferrozine reagent (Fluka, 97%) and ferrous sulfate heptahydrate (Vetec, 99%) were used for the FIC assay. Ascorbic acid (Vetec, 99%), ethylenediaminetetracetic acid (EDTA; Vetec, 99%), tannic acid (Vetec, 99%) and rutin (Acros Organics, 97%) were used as standards. Weights were measured on a Shimadzu analytical balance (AX200), and absorbance readings were recorded using a Shimadzu UV-Vis (UV mini-1240) spectrophotometer.

### 3.3. Preparation of Extracts

The samples were dried in an oven (Nova Técnica NT-503) for three days at 45 ± 5 °C, powdered in a Willy vertical mill grinder (Adamo 340) and standardized on sieves, resulting in a 20-mesh particle size (1.2 mm). The samples were extracted by maceration for 72 h with 70% ethanol (500 mg/mL) and then filtered through 9 mm filter paper. The extracts were subjected to slow evaporation (Fisatom 801) under reduced pressure at a temperature of 40 ± 5 °C. Yields varied between 11.54% and 26.78% (Table 2).

### 3.4. Determination of the Phenolic Content

#### 3.4.1. Total Phenolic Content (TPC) and Total Tannin Content (TTC)

We used the Folin-Ciocalteu method to determine the TPC. To determinate the residual phenolic content, we used the casein precipitation method followed by the Folin-Ciocalteu method, where the TTC was the difference between the total and residual phenolic content [45,46]. To quantify the TPC, diluted extract in methanol (2 mL, 0.5 mg/mL) was mixed with an aqueous solution of Folin-Ciocalteu (5 mL, 10%, v/v), Na_2_CO_3_ (10 mL, 75 mg/L) and distilled water (84 mL). The solution was allowed to stand in the dark for 30 min. The absorbance was measured at 760 nm using distilled water to reset the equipment. To quantify the residual phenolic content, the extract (15 mL) was agitated for 3 h with casein (1 g) and then filtered and diluted to 25 mL with distilled water. The residual phenolic content was determined from 5 mL of the filtrate using the Folin-Ciocalteu method. All procedures were performed with six replicates. The correlation equation constructed with tannic acid (0.1 to 6 µg/mL) was y = 0.0616x + 0.0051 (R2 = 0.9986). The TPC and TTC were expressed as milligrams of tannic acid equivalents per gram of dry extract (mg TAE/g).

#### 3.4.2. Total Flavonoid Content (TFC)

The content of flavonoids in the extracts was determined using the method described by Peixoto Sobrinho *et al*. [47]. Aliquots of extract diluted in methanol (1 mL, 0.5 mg/mL) were mixed with glacial acetic acid (0.6 mL), a methanol solution of pyridine (10 mL, 20%, v/v), a methanol solution of aluminum chloride (2.5 mL, 50 mg/L) and distilled water (10.9 mL). The solution was allowed to stand in the dark for 30 min. The solution absorbance was measured at 420 nm using distilled water to reset the equipment. All procedures were performed with six replicates. The correlation equation constructed with rutin (2 to 50 µg/mL) was y = 0.0268x + 0.0101 (R² = 0.9996). The TFC was expressed as milligrams of rutin equivalent per gram of dry extract (mg RE/g).

### 3.5. Evaluation of Antioxidant Activity

#### 3.5.1. Radical Scavenging Activity (DPPH Assay)

The DPPH assay was performed in triplicate based on the method described by Sousa *et al*. [48]. Different concentrations of each CEE or standard (25-250 µg/mL) in the amount of 0.5 mL were added to the DPPH methanolic solution (3 mL, 40 µg/mL w/v). The solution was allowed to stand for 30 min in the dark, and the absorbance was measured at 517 nm. Analyses were performed using methanol to reset the spectrophotometer. Measurements were compared with a negative control consisting of DPPH methanolic solution (40 µg/mL w/v) and were used as blank concentrations for each CEE or standard (0.5 mL in 3 mL of methanol). The DPPH assay results were calculated from a calibration curve to obtain the percentage of antioxidant activity (Equation 1) versus the concentrations of CEE or standard. The data are represented as IC_50_ values, the concentration of sample required to reduce the absorbance of the negative control by 50%:

%AA = 1 – (Abs_sample_ – Ab_sblank_ / Abs_negative control_) × 100 (1)

The results are also expressed as the ascorbic acid equivalent antioxidant capacity (AEAC) as calculated by Equation 2. The IC_50_ of ascorbic acid was 16.12 ± 0.01 µg/mL:

AEAC (mg AA / 100 g) = (IC_50 ascorbic acid_ / IC_50 sample_) × 100 (2)

#### 3.5.2. Chelating Assay (FIC)

The FIC assay was performed in triplicate as described by Chew *et al*. [16]. Different 1 mL dilutions of each CEE (1-7 mg/mL) or EDTA (10-100 µg/mL) were mixed with FeSO_4_ (1 mL, 0.1 mM, w/v), followed by ferrozine (1 mL, 0.25 mM, w/v). The solution was allowed to stand for 10 minutes in the dark, and the absorbance was measured at 562 nm. Analyses were performed using methanol to reset the spectrophotometer. Measurements were compared with a negative control consisting of methanol (1 mL, 75%, v/v), FeSO_4_ (1 mL) and ferrozine (1 mL). The blank samples consisted of 1 mL of the dilutions of the samples with FeSO_4_ solution (2 mL). The capacity of the sample to chelate ferrous ions was calculated from a calibration curve to obtain the percentage of chelating activity (Equation 3) versus concentrations of CEE or standard, and the data are expressed as IC_50_:

%QA = 1 – (ABS_sample_ – ABS_blank_ / ABS_negative control_) × 100 (3)

### 3.6. Statistical Analysis

The Kolmogorov-Smirnov test was used to confirm the normality of the data, and parametric tests were used for analysis of variance and correlation. We analyzed the variance using ANOVA followed by Tukey’s test. The Pearson correlation test was used to compare the TPC, TTC, and TFC and the IC_50_ of the samples. Differences were considered statistically significant at p < 0.05. BioEstat 5.0 was used to perform statistical analysis and *GraphPad Prism* 5 was used for regression analysis and to generate graphs.

## 4. Conclusions

Aligning traditional knowledge with scientific assessment through laboratory tests, *C. pyramidalis* and *S. glandulosum* represent good candidates for the crossover from folk medicine to modern therapeutics based on their phenolic content and antioxidant activity. In this sense, extract from the leaves of *C. pyramidalis* could be a viable alternative for obtaining compounds with great commercial potential, given its high antioxidant potential and renewability. This compound could provide new chelating agents for the chemical, food and pharmaceutical industries.
